# miR-199a-5p inhibits aortic valve calcification by targeting ATF6 and GRP78 in valve interstitial cells

**DOI:** 10.1515/med-2023-0777

**Published:** 2023-08-31

**Authors:** Heng Chu, XingLi Fan, Zhe Zhang, Lin Han

**Affiliations:** Department of Thoracic Surgery, Qingdao Hospital, University of Health and Rehabilitation Sciences (Qingdao Municipal Hospital), Qingdao, Shandong, 266000, China; Department of Cardiovascular Surgery, Changhai Hospital Affiliated to Naval Medical University, Shanghai 200433, China; Department of Thoracic Surgery, Qingdao Hospital, University of Health and Rehabilitation Sciences (Qingdao Municipal Hospital), No. 1 Jiaozhou Road, Shibei District,, Qingdao, Shandong, 266000, China; Department of Cardiovascular Surgery, Changhai Hospital Affiliated to Naval Medical University, 168 Changhai Road, Yangpu District, Shanghai 200433, China

**Keywords:** miR-199a-5p, calcific aortic valve disease, valvular interstitial cells, 78 kDa glucose-regulated protein, activating transcription factor 6, endoplasmic reticulum stress

## Abstract

Calcific aortic valve disease (CAVD) is an important cause of disease burden among aging populations. Excessive active endoplasmic reticulum stress (ERS) was demonstrated to promote CAVD. The expression level of miR-199a-5p in patients with CAVD was reported to be downregulated. In this article, we aimed to investigate the function and mechanism of miR-199a-5p in CAVD. The expression level of miR-199a-5p and ERS markers was identified in calcific aortic valve samples and osteogenic induction by real-time quantitative polymerase chain reaction (RT-qPCR), immunohistochemistry, and western blotting (WB). Alizarin red staining, RT-qPCR, and WB were used for the verification of the function of miR-199a-5p. The dual luciferase reporter assay and rescue experiment were conducted to illuminate the mechanism of miR-199a-5p. In our study, the expression level of miR-199a-5p was significantly decreased in calcified aortic valves and valve interstitial cells’ (VICs) osteogenic induction model, accompanying with the upregulation of ERS markers. Overexpression of miR-199a-5p suppressed the osteogenic differentiation of VICs, while downregulation of miR-199a-5p promoted this function. 78 kDa glucose-regulated protein (GRP78) and activating transcription factor 6 (ATF6), both of which were pivotal modulators in ERS, were potential targets of miR-199a-5p. miR-199a-5p directly targeted GRP78 and ATF6 to modulate osteoblastic differentiation of VICs. miR-199a-5p inhibits osteogenic differentiation of VICs by regulating ERS via targeting GRP78 and ATF6.

## 1 Introduction

Calcific aortic valve disease (CAVD) is an important cause of disease burden in the elderly throughout the world and is characterized by the irreversible calcification of valve leaflets. With the disease progression, severe aortic valve stenosis and cardiac failure eventually lead to death. Unfortunately, no medical therapies, especially drug treatments, have been proven to be effective in preventing, delaying, or even reversing disease progression [1,2]. It follows that the prognosis of symptomatic patients is poor. Therefore, aortic valve replacement assisted with extracorporeal circulation or transcatheter aortic valve implantation is available treatment method. CAVD is an active disease regulated by lipoprotein deposition, oxidation, inflammation, and osteoblastic differentiation of valve interstitial cells (VICs). Improving the understanding of the underlying mechanisms of CAVD is beneficial to the development of future treatments.

Endoplasmic reticulum stress (ERS) is a cellular response resulting from those unfolded or misfolded proteins abnormally accumulating in its lumen, which may lead to apoptosis and autophagy. Endoplasmic reticulum proteostasis is regulated by the unfolded protein response (UPR), which includes three downstream signal transduction pathways: PERK, IRE1/Xbp1s, and activating transcription factor 6 (ATF6). The 78 kDa glucose-regulated protein (GRP78), an essential endoplasmic reticulum resident protein chaperone, interacts with the luminal domains of the three transmembrane sensors and anchors them to the surface of the endoplasmic reticulum membrane. ERS activation or depression plays an essential role in many pathophysiological conditions, including obesity, atherosclerosis, tumors, myocardial infarction, and diabetes mellitus. Evidence illustrated that ERS activation serves as a good model in the development of CAVD via reducing histone deacetylase 6 [3]. Another study showed that tauroursodeoxycholic acid – a sort of ERS inhibitor – attenuates aortic valve calcification in the hypercholesterolemic rabbit and mouse models [4]. Recently, a study revealed that in the absence of ERS, downstream effector leads to the protection and prevention of aortic valve calcification [5]. Obviously, overactive ERS has been demonstrated to promote CAVD.

microRNA is a non-coding RNA with post-transcriptional regulatory function, and its length is about 18–24 bp. miR-199a/214 cluster is essential for embryonic development and is very common in vertebrates, as described previously [6]. Previous studies have highlighted that miR-214 is a central regulator of valvular calcification [7]. For instance, it has been reported that the overexpressed miR-214 was verified as an inhibitor suppressing valvular calcification in VICs [8]. Likewise, miR-214 related to the excessive inflammatory reaction was an essential link in the calcification process in CAVD [9]. Additionally, compared with those in patients with rheumatic valvular heart disease, miR-199a-5p was evidently reduced in CAVD people, suggesting that a decreased expression of miR-199a-5p may be related to the progression of CAVD [10]. However, the potential mechanism of how miR-199a-5p affects CAVD is not clear. Thus, in the present study, the purpose was to determine the function and regulatory mechanism of miR-199a-5p in CAVD.

## 2 Materials and methods

### 2.1 Samples

During heart valve replacement surgery, the calcific aortic valve leaflets were obtained from 21 patients diagnosed with aortic valve stenosis. Patients under 65 years of age were excluded. The valve leaflets obtained from 14 heart transplantation recipients suffering from dilated cardiomyopathy served as the control group (Table A1). All human valve samples were instantly resected in surgeries and stored in a −80°C freezer.


**Ethics approval and consent to participate:** The present study was approved by the Ethic Committee of Changhai Hospital, Naval Medical University (No. CH20180630). Patients whose valve leaflets were collected provided written informed consent.
**Patient consent for the use of valve leaflets:** All patients provided written informed consent for the scientific use of valve leaflets.

### 2.2 Isolation and culture of primary VICs

The aortic valve leaflets in the control group collected from the heart transplantation recipients were soaked into phosphate-buffered solution (PBS). The valvular endothelial cells (VECs) on the sample surface were eliminated by 0.2% collagenase II on a shaker for 15 min at 37°C. The tissue was cut into small pieces, and then, digestion with the aforementioned collagenase II was performed for 2 h. Primary VICs were cultured in a mixed medium including Dulbecco’s modified Eagle’s medium, 1% penicillin and streptomycin, and 10% fetal bovine serum. The third to fifth passages of VICs were used for further experiments.

### 2.3 Differentiation of VICs into osteoblasts

VICs were induced to differentiate into osteoblasts by osteogenesis medium, which is a complete medium containing sodium dihydrogen phosphate dihydrate (2 mmol/L), ascorbic acid (50 μg/mL), and insulin (10^−7^ mol/L). The osteogenesis medium was replaced every 2 days.

### 2.4 Transfection

The downregulation and upregulation of miR-199a-5p in VICs were produced by transfecting with 100 pmol miR-199a-5p agomiR or antagomiR (GenePharma, China) individually by lipofectamine 2000 (Thermo, USA) in accordance with protocol. To knockdown GRP78 or ATF6 in VICs, cells were transfected with GRP78 or ATF6 siRNA (Obio Technology, China). 8 h ahead of transfection, the medium was changed with osteogenic differentiation medium.

### 2.5 Real-time quantitative polymerase chain reaction (RT-qPCR)

The miRNAs, mRNAs, and total RNA were extracted by TRIzol (Invitrogen, USA) under the guidance of protocol. The PrimeScript RT reagent Kit (TaKaRa, Japan) was applied to the synthesis of cDNA. miRNA First-Strand Synthesis Kit (TaKaRa, Japan) was used for reverse-transcribed microRNAs. TB Green RT-PCR Kit and Light Cycler 480 System were employed for quantitative real-time PCR assay. The amplification protocol was as follows: pre-denaturation at 95°C for 5 min, denaturation at 95°C for 30 s, annealing at 62°C for 30 s, and extension at 72°C for 30 s. The cycle value was set as 40. At last, the expressions of mRNA and microRNA were normalized to the reference gene GAPDH and U6, respectively. Results were analyzed using the 2^−△△Ct^ method. All primers are summarized in Table 1.

**Table 1:** Primer sequence

Gene	Primer sequence
hsa-Runx2-Forward	CTGTGGTTACTGTCATGGCG
hsa-Runx2-Reverse	AGGTAGCTACTTGGGGAGGA
hsa-GRP78-Forward	CTATTGGGGTGTTTCGCGAG
hsa-GRP78-Reverse	GAGAGCTTCATCTTGCCAGC
hsa-ATF6-Forward	CTGTTACCAGCTACCACCCA
hsa-atf6-Reverse	GGGAGCCAAAGAAGGTGTTG
hsa-ATF4-Forward	CACCGCAACATGACCGAAAT
hsa-ATF4-Reverse	TACCCAACAGGGCATCCAAG
hsa-GAPDH-Forward	GCTCAACGTGTGGTCATCTC
hsa-GAPDH-Reverse	ACCCTTCCACGATCCCAAAT
hsa-miR-199a-5p-Forward	CCCAGTGTTCAGACTACCTGTTC

### 2.6 Alizarin red staining

Alizarin red staining was conducted to the identification of calcium deposition after the osteogenic differentiation procedure in a period of 7 days. In brief, after being washed twice with PBS, VICs were fixed by 75% ethyl alcohol for 15 min, and then, VICs were washed twice again. Finally, the Alizarin red solution (Servicebio, China) stained VICs for 10 min. For the removal of non-specific staining, cells were further washed with 95% ethanol. The positive stain photos were evaluated and taken by the digital microscope. The quantification of the Alizarin red stain was carried out by measuring the absorbance of the supernatant.

### 2.7 Dual-luciferase reporter assay

The wild type (WT) of GRP78 and ATF6 3′-UTR which contained the predicted binding site of miR-199a-5p was inserted into the psiCHECK-2 vector (Sangon Biotech, China) individually. Cotransfection of HEK293T cells with luciferase reporter plasmid and miR-199a-5p agomiR or agomiR-NC was performed by Lipofectamine 2000 (Thermo, USA). The firefly and renilla luciferase activities were determined by a Dual-luciferase reporter assay system (Promega, USA).

### 2.8 Western blotting (WB)

The cellular protein was extracted with SDS buffer. Cell lysates were separated by 10% SDS-PAGE and transferred onto polyvinylidene difluoride membranes. After the blocking procedure, the membrane was stained with several primary antibodies followed by corresponding secondary antibodies (Jackson Immuno Research; 1:5,000 dilution). Protein bands on membranes were detected by an ECL kit (Thermo). The relevant primary antibodies applied above were GAPDH (Protein tech; 60004-1-Ig; 1:5,000 dilution), Runx2 (CST; # 12556; 1:500 dilution), GRP78 (Proteintech; 11587-1-AP; 1:800 dilution), ATF6 (Abcam; ab203119; 1:800 dilution), ATF4 (Santa Cruz; sc-390063; 1:50 dilution), and XBP1 (Abcam; ab37152; 1:800 dilution).

### 2.9 Immunohistochemistry

These aortic valve specimens were made into paraffin slides. After that, deparaffination and hydration were performed before the blocking procedure. Subsequently, these slides were incubated with primary antibodies separately, including ATF4 (Affinity; DF6008; 1:100 dilution), CHOP (Affinity; DF6025; 1:100 dilution), GRP78 (Proteintech; 11587-1-AP; 1:200 dilution), Runx2 (Abcam; ab23981; 1:200 dilution), ATF6 (Abcam; ab203119; 1:200 dilution), and XBP1 (Abcam; ab37152; 1:200 dilution). Nuclei were counterstained with hematoxylin. In the next stage, the positive staining was detected by an UltraSensitive SP IHC Kit and a DAB Kit (Maixin Biotech; KIT-9710; China).

### 2.10 Immunofluorescence

The fixations of VICs were carried out with 4% paraformaldehyde. Triton X-100 (0.5%) was used for permeabilization. Primary antibodies from Vimentin (Santa Cruz; 1:1,000 dilution), α-SMA (Affinity; 1:1,000 dilution), to CD31 (Abcam; 1:50 dilution) were incubated overnight at 4°C on a shaker. Next, relevant secondary antibodies labeled with Cy3 (Servicebio; 1:500 dilution) were incubated for 1 h at RT. The nuclei were stained with 4′,6-diamidino-2-phenylindole (Servicebio; 1:500 dilution). Fluorescent images were captured using an inverse fluorescent microscopy.

### 2.11 Statistical analysis

The statistical analyses were carried out by IBM SPSS v. 21.0 and GraphPad Prism software. Mean values and standard deviation were described for continuous variables. Shapiro–Wilk test was used for normality analysis. Student’s *t*-test and one-way analysis of variance were used for analysis between two groups and multiple comparisons separately. *p* values <0.05 were considered statistically significant for all tests.

## 3 Results

### 3.1 Downregulation of miR-199a-5p in calcified aortic valve and VICs’ osteogenic induction model

**Figure 1:**
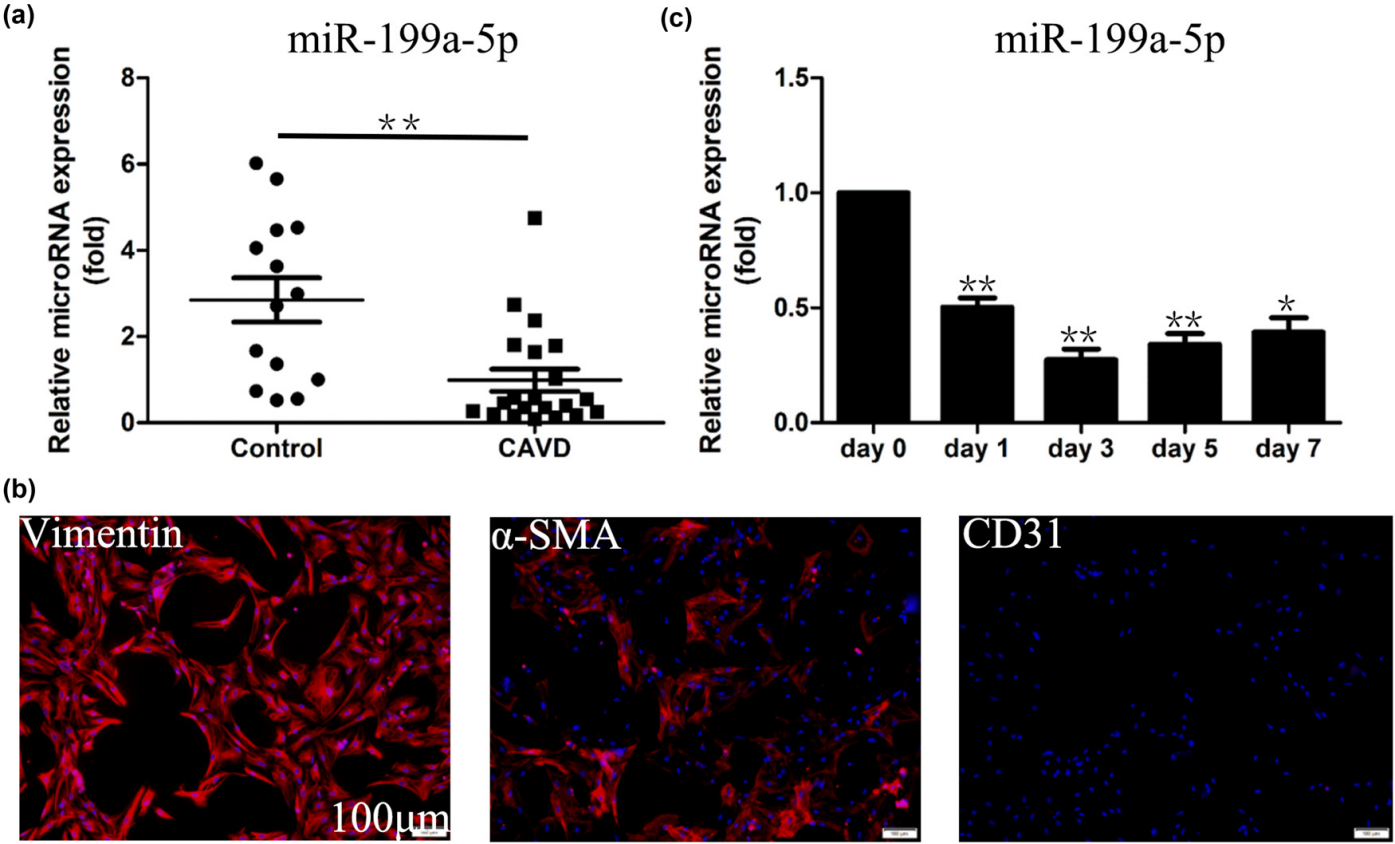
Downregulation of miR-199a-5p in calcified aortic valve and valve interstitial cells (VICs) osteogenic induction model. (a) RT-qPCR analysis of miR-199a-5p in the calcific aortic valve. The miR-199a-5p level dramatically decreased among the CAVD samples. (b) The expression of Vimentin and CD31 in VICs. Scale bar, 100 μm. (c) RT-qPCR results of miR-199a-5p in VICs after osteogenic induction. The miR-199a-5p level dramatically decreased on day 1. *p < 0.05, **p < 0.01.

To investigate whether miR-199a-5p was associated with the pathological process of CAVD, we first analyzed the expression level of miR-199a-5p in calcified aortic valves. Our results demonstrated that the miR-199a-5p expression in calcified aortic valves was significantly lower than that of normal valves (Figure 1a). The normal aortic valve is composed of VICs colonizing in the interstitial substance, VECs cover the surface of the valve leaflets and the extracellular matrix. The results of immunofluorescence staining were a combination of vimentin-positive staining and CD31-negative staining, demonstrating that these cells isolated and cultured from the normal aortic valves were pure VICs, rather than compounds containing VECs (Figure 1b). Then, the VICs were stimulated by the osteogenic induction medium containing inorganic phosphate.

We then used the VICs’ osteogenic induction model to identify the effect of miR-199a-5p in the osteogenic differentiation of VICs. miR-199a-5p expression was dramatically reduced to a low level from day 1 to day 7 (Figure 1c). These results, taken together, enable us to support our hypothesis that miR-199a-5p deficiency plays a prominent role in CAVD progression.

### 3.2 ERS was upregulated in VICs’ osteogenic induction model and calcified aortic valve

**Figure 2:**
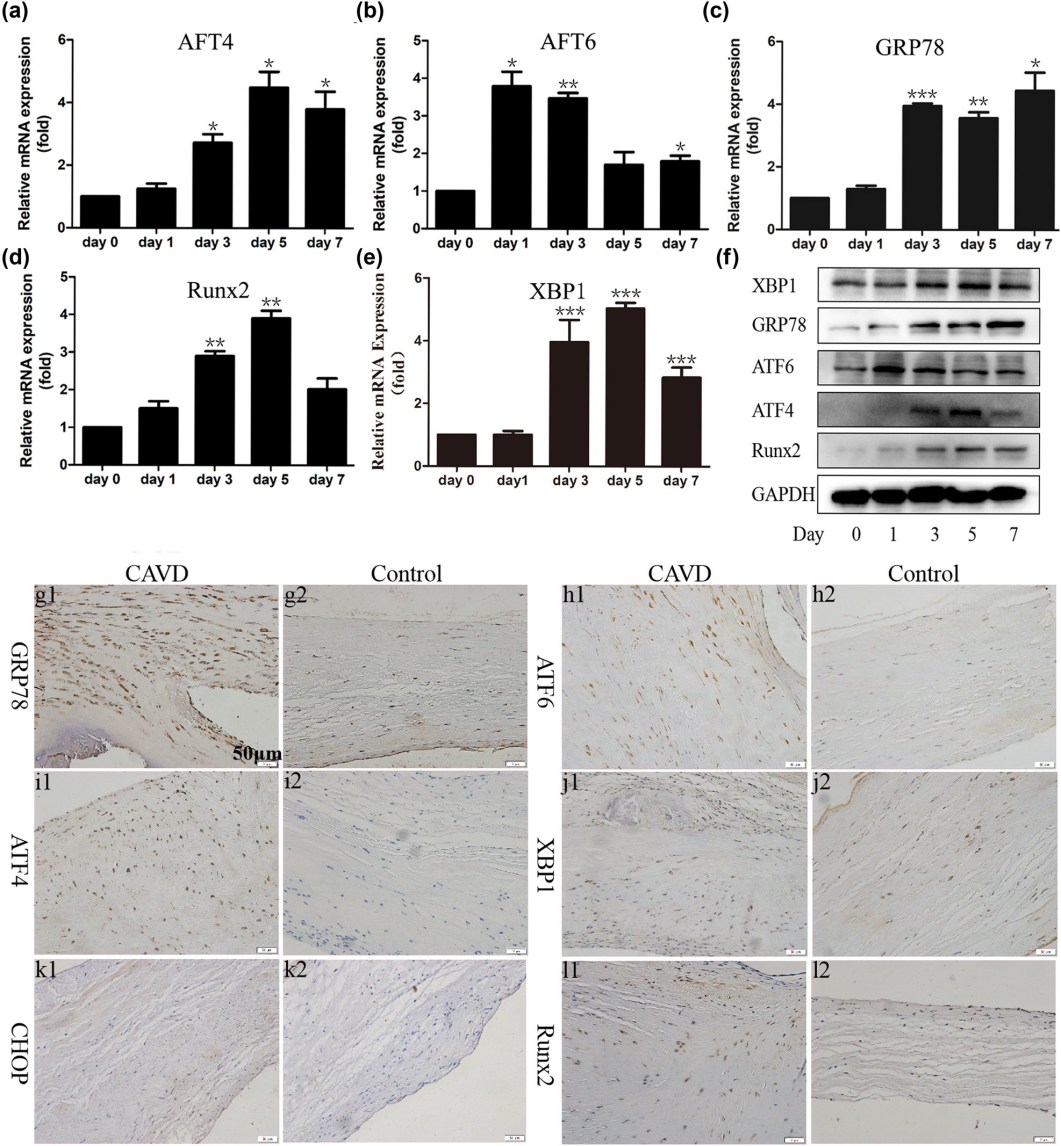
ERS was upregulated in VICs’ osteogenic induction model and calcified aortic valve. (a–e) RT-qPCR analysis of GRP78, ATF6,ATF4, XBP1, and Runx2 of VICs after osteogenic induction. The mRNA level significantly increased on the third day. (f) WB of GRP78, ATF6, ATF4, XBP1, and Runx2 of VICs. The protein level significantly increased on the third day. Representative immunohistochemical staining of ERS-related proteins (g) GRP78, (h) ATF6, (i) ATF4, and (j) XBP1 in the aortic valve. (k) Representative immunohistochemical staining of CHOP. The expression of CHOP was hardly detected in the two groups. (l) Representative immunohistochemical staining of Runx2. The Runx2 level was upregulated in the CAVD sample. Scale bars, 50 μm. *p < 0.05, **p < 0.01, ***p < 0.001.

The RT-qPCR results indicated that, on the third day after osteogenic induction, the mRNA levels of ERS-related proteins from GRP78, ATF6 to ATF4 were remarkably increased (Figure 2a–c), suggesting that the activated ERS may be involved in the process of osteogenic differentiation of VICs. The Runx2 and XBP1 were also increased on the third day after osteogenic induction (Figure 2d and e). An identical tendency was observed by WB (Figure 2f). The protein expression levels of GRP78, ATF6, ATF4, XBP1, and Runx2 were upregulated on the third day.

The previous studies have confirmed that overactive ERS participated in aortic valve calcification. Immunohistochemistry staining showed that compared to those in the control group, the expression of GRP78, ATF6, ATF4, and XBP1 in the CAVD group was significantly upregulated (Figure 2g–j). However, CHOP expression was hardly detected in both the CAVD and control groups (Figure 2k). Additionally, the expression level of Runx2, which was a pivotal osteogenic-related protein, was also significantly upregulated (Figure 2l). These results demonstrated that the activated ERS may be related to the pathological process of CAVD.

### 3.3 miR-199a-5p suppressed osteoblastic differentiation of VICs

**Figure 3:**
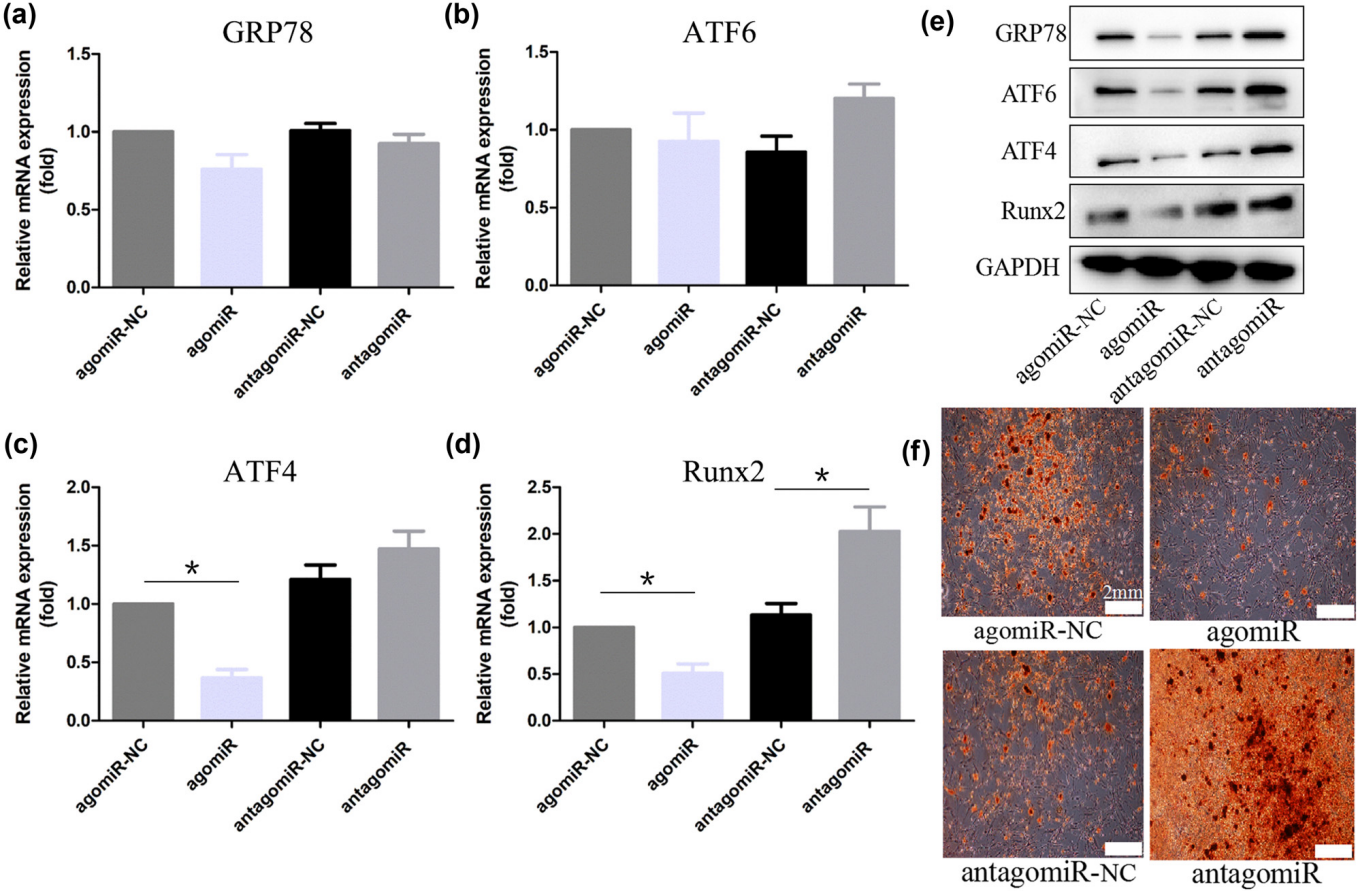
miR-199a-5p attenuates osteoblastic differentiation of VICs *in vitro*. (a–d) RT-qPCR analysis of GRP78, ATF6, ATF4, and Runx2 of VICs after transfection of agomiR-199a-5p and antagomiR-199a-5p. (e) The protein levels of GRP78, ATF6, ATF4, and Runx2. (f) Representative Alizarin red staining of calcium deposition. Scale bars, 2 mm. *p < 0.05.

For the verification of the inhibition effect of miR-199a-5p in the osteogenic differentiation of VICs, the miR-199a-5p in VICs was overexpressed and knocked down by transfecting agomiR-199a-5p and antagomiR-199a-5p, separately. The RT-qPCR analyses revealed that the mRNA levels of GRP78 and ATF6 did not change significantly (Figure 3a and b). Moreover, the mRNA levels of Runx2 and ATF4 were decreased with agomiR-199a-5p transfection. Conversely, silencing miR-199a-5p promoted the expression level of Runx2 (Figure 3c and d). Besides, the WB analysis showed that the protein expressions of GRP78, ATF6, ATF4, and Runx2 were inhibited by agomiR-199a-5p transfection. In contrast, they were increased by antagomiR-199a-5p transfection (Figure 3e). Similarly, the mineralization deposition was suppressed by transfecting agomiR-199a-5p while accelerated by transfecting antagomiR-199a-5p as evidenced by Alizarin red staining (Figure 3f). Taken together, miR-199a-5p attenuated osteogenic differentiation of VICs *in vitro*.

### 3.4 miR-199a-5p directly targeted at ATF6 and GRP78

**Figure 4:**
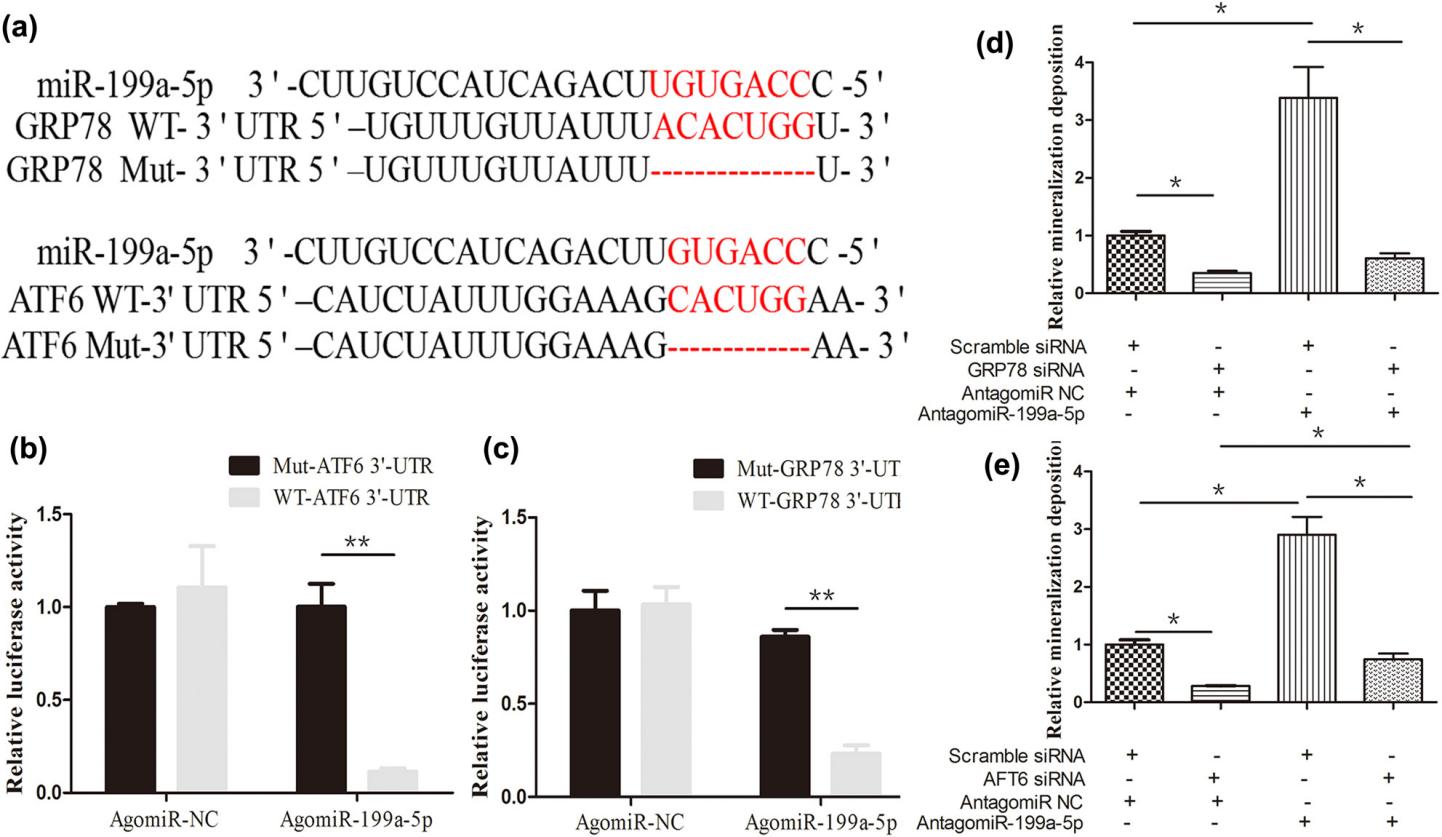
miR-199a-5p directly target ATF6 and GRP78. (a) Schematic illustration of the putative binding site (highlighted in red) of miR-199a-5p. The luciferase activity of WT-GRP78-3′-UTR (b) and WT- ATF6-3′-UTR (c) was remarkably suppressed by the transfection of agomiR-199a-5p. (d) Downregulation of GRP78 significantly suppressed the calcium deposition of VICs. (e) Downregulation of ATF6 dramatically alleviated the calcium deposition of VICs. WT, wild type. *p < 0.05, **p < 0.01.

The potential molecular mechanism of miR-199a-5p in regulating the osteogenic differentiation of VICs was further analyzed. ATF6 and GRP78 were selected for further analysis (Figure 4a). At first, the luciferase reporter plasmid vector, which contained ATF6, GRP78 3′-UTR, and corresponding mutation vector, was constructed. Second, miR-199a-5p overexpression evidently reduced the luciferase activity of WT-ATF6 3′-UTR and WT-GRP78 3′-UTR (Figure 4b and c). However, the vector that contained relevant mutant sequence eliminated this inhibitory effect, indicating that miR-199a-5p specifically targeted ATF6 and GRP78.

At last, to investigate whether ATF6 and GRP78 were essential for miR-199a-5p to suppress the osteogenic differentiation of VICs, the rescue experiments were performed. The results of rescue experiments presented that the calcium deposition was obviously weakened after si-GRP78 transfection. In contrast, the mineralization deposition was aggravated by antagonmiR-199a-5p transfection. Co-transfection with si-GRP78 eliminated the aggravating mineralization deposition induced by antagomiR-199a-5p (Figure 4d). Similarly, co-transfection with si-ATF6 also alleviated the effect of the aggravating mineralization deposition induced by antagomiR-199a-5p (Figure 4e). To sum up, we confirmed that GRP78 and ATF6 were the functional targets of miR-199a-5p in VICs’ osteogenic differentiation.

## 4 Discussion

CAVD is one of the most common cardiovascular diseases among the elderly. However, no effective methods were found to prevent and treat it until present times. Our research offers a new insight into the prevention and treatment of CAVD in the future. In this study, we found a novel role of miR-199a-5p in modulating osteoblastic differentiation of VICs. It functions by regulating ERS via targeting GRP78 and ATF6.

The imbalance of miR-199a-5p has been proved to be related to many diseases. For example, Savary et al. proved that miR-199a-5p produced by DNM3OS was able to prevent pulmonary fibrosis [11]. Another study displayed that miR-199a-5p in liver cancer cells might exert inhibiting function on cell proliferation and tumorigenesis by interfering with HK2 expression [12]. Additionally, evidence showed that a high level of miR-199a-5p indicated poor survival as well as a high recurrence rate in prostate cancer patients [13]. A previous report revealed that miR-199a-5p negatively regulated macrophage-mediated inflammation, demonstrating its crucial role in diabetes mellitus [14]. On top of them, the protection effect of miR-199a-5p in ischemia/reperfusion of myocardium and cardiac remodeling has also been clarified as follows. Several studies suggested that the absence of miR-199a-5p facilitated lots of downstream target genes, then alleviating cytotoxicity in hypoxic cardiomyocytes [15,16]. Furthermore, miR-199a-5p was documented to exert its function in mediating cardiomyocyte apoptosis via targeting JunB [17]. A recent study revealed that knockdown of miR-199a-5p in cyanotic congenital heart disease was conducive to cardiomyocytes against hypoxia-induced ERS [18]. Nevertheless, the function and mechanism of miR-199a-5p in valvular heart disease need to be further elucidated. Asulin et al. demonstrated that miR-199a-5p in the CAVD group decreased via analyzing the microRNA expression profile in either calcific or rheumatic valve samples [10]. In the current study, the RT-qPCR analyses verified the miR-199a-5p deficiency in calcific valve specimens. Silencing of miR-199a-5p facilitated osteogenic differentiation of VICs *in vitro*, illustrating that miR-199a-5p may participate in the CAVD process.

UPR includes three classic signaling pathways: PERK, IRE1/XBP1, and ATF6. GRP78, an ER-resident protein chaperone, binds to the above three proteins and anchors them on the ER lumen membrane to maintain the quiescent state of UPR. Our immunohistochemistry studies showed that the expression levels of GRP78 and ATF6 in the CAVD group were strikingly upregulated, indicating that excessive active ERS may be related to the pathological process of CAVD.

The expression of GRP78 induced by ischemia/reperfusion in cardiomyocytes could stimulate Akt signaling and protect cells from oxidative damage and apoptosis [19,20]. Meanwhile, Wang et al. found that the embryos that a lack of GRP78 specifically in cardiomyocytes exhibited cardiovascular malformations, and GRP78 is essential for maintaining cardiac contractility and function [21,22]. ATF6, another crucial transcriptional regulator of ER proteostasis, was also reported to facilitate myocardial ischemia/reperfusion injury [23,24]. Jin et al. verified that ATF6 overexpression mitigated the reactive oxygen species and necrotic cell death in cardiomyocytes. Both were caused by silencing ATF6 [25]. On the other hand, ATF6 was demonstrated to suppress the activation of cardiac fibroblasts induced by transforming growth factor β, which leads to reducing cardiac fibrosis [26]. Additionally, our results of RT-qPCR and WB analyses displayed that GRP78 and ATF6 were dramatically increased on the third day after osteogenic induction, promoting this assumption that the activated ERS may be involved in the process of osteogenic differentiation of VICs.

Non-coding RNA has been widely documented to regulate the valvular remodeling processes in CAVD [7]. Among the variously reported microRNAs, several studies have highlighted miR-214 and miR-204 as pivotal regulators of osteogenic differentiation of VICs. Li et al. testified that miR-214-3p could inhibit CAVD by targeting two core osteogenic transcription factors: Osterix/Sp7 and ATF4 [8]. Additionally, miR-204 deficiency in VICs enhanced valvular osteogenic activity by promoting the expression of Runx2 and Smad4 [27–30]. Toshima et al. demonstrated that miR-34a promoted valve calcification by regulating the Notch1–Runx2 axis. [31] The bicuspid aortic valve malformation patients shared higher risks of CAVD when compared to the patients with tricuspid aortic valve leaflets, accompanying the remarkably lower expression level of miR-195. The downregulation of miR-195 was related to valvular calcification by targeting SMAD7, which promoted the remodeling of the extracellular matrix [32].

In the current study, we found that compared with those increased by antagomiR-199a-5p transfection, the protein expressions of GRP78 and ATF6 in VICs were inhibited by agomiR-199a-5p transfection. Similarly, the calcium deposition was accelerated by silencing miR-199a-5p while inhibited by overexpressing miR-199a-5p as evidenced by Alizarin red staining. These further verified that miR-199a-5p attenuated the osteogenic differentiation of VICs *in vitro*. Finally, our rescue experiments proved that GRP78 and ATF6 were the functional targets of miR-199a-5p. In general, our results above demonstrate that miR-199a-5p inhibits osteogenic differentiation of VICs by suppressing GRP78 and ATF6.

## Appendix

**Table A1:** Patient profiles

Variables	CAVD (*n* = 21)	Control (*n* = 14)	*p* Value
Age, years	70.4 ± 3.9	53.6 ± 11.5	<0.001***
Male, *n* (%)	16 (76.2)	10 (71.4)	0.752
BMI, kg/m²	23.3 ± 3.6	23.6 ± 3.9	0.834
LVEF, %	52.9 ± 13.4	20.6 ± 7.2	<0.001***
**Medical History, *n* (%)**			
Hypertension	13 (61.9)	0 (0)	<0.001***
Diabetes mellitus	2 (9.5)	1 (4.8)	1
**Preoperative medication, *n* (%)**			
ACEI	8 (38.1)	9 (64.3)	0.129
BB	7 (33.3)	10 (71.4)	0.027
CCB	2 (9.5)	0 (0)	0.506

CAVD, calcific aortic valve disease; BMI, body mass index; LVEF,left ventricle ejection fraction; ACEI, angiotensin converting enzyme inhibitors; BB, beta-blockers; CCB, calcium-channel blockers.

*p* < 0.001.
